# Chitosan‐Functionalized Recycled Polyethylene Terephthalate Nanofibrous Membrane for Sustainable On‐Demand Oil‐Water Separation

**DOI:** 10.1002/gch2.202000107

**Published:** 2021-01-12

**Authors:** Andrea Baggio, Hoan N. Doan, Phu P. Vo, Kenji Kinashi, Wataru Sakai, Naoto Tsutsumi, Yasuro Fuse, Marco Sangermano

**Affiliations:** ^1^ Master's Program of Innovative Materials Kyoto Institute of Technology Matsugasaki, Sakyo Kyoto 606‐8585 Japan; ^2^ Master's Program of Materials Engineering Politecnico di Torino Corso Duca degli Abruzzi 24 Torino 10129 Italy; ^3^ Doctor's Program of Materials Chemistry Kyoto Institute of Technology Matsugasaki, Sakyo Kyoto 606‐8585 Japan; ^4^ Faculty of Materials Science and Engineering Kyoto Institute of Technology Matsugasaki, Sakyo Kyoto 606‐8585 Japan; ^5^ Center of Environmental Science Kyoto Institute of Technology Matsugasaki, Sakyo Kyoto 606‐8585 Japan; ^6^ Department of Applied Science and Technology (DISAT) Politecnico di Torino Corso Duca degli Abruzzi 24 Torino 10129 Italy

**Keywords:** chitosan, electrospinning, oil‐water separation, polyethylene terephthalate, waste recycling

## Abstract

The preservation of marine ecosystems is one of the most severe challenges at present. In particular, oil‐water separation from oil spills and oily wastewater is important. For this reason, a low‐cost, effective, and sustainable polymeric solution is in high demand. In this work, a controlled‐wettability membrane for selective separation of oil‐water mixtures and emulsions is developed. The nanofibrous membrane is prepared via a facile and cost‐effective electrospinning technique using environmentally sustainable materials, such as recycled polyethylene terephthalate and chitosan. The effect of different concentrations of chitosan on the morphology, chemical composition, mechanical properties, wettability, and separation performance of the membrane is evaluated. The membranes exhibited underoil superhydrophobic and underwater superoleophobic behavior, which is essential to perform the selective separation. In fact, the designed filter has competitive antifouling properties (oil intrusion pressure > 45 kPa) and showed high heavy‐ and light‐oil/water separation efficiencies (>95%) both for emulsions and immiscible mixtures.

## Graphical abstract

Controlled‐wettability membranes for selective separation of oil‐water mixtures and emulsions are fabricated using environmentally sustainable recycled polyethylene terephthalate (r‐PET) and chitosan based electrospun nanofibers.

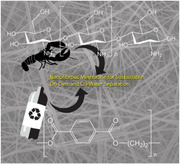

## 1 Introduction

In recent decades, increasing attention has been paid to oily wastewater and polluted oceanic waters, which, along with others, represent some of the main current problems.^[^ ^1^ ^]^ Although the occurrence of petrol disasters is gradually decreasing, other related dispersed oil sources are causing oil spills, including regular shipping operations, municipal and industrial effluents, and oil rig operations.^[^ ^2^ ^]^ Many solutions have been developed over the years, such as API oil‐water separators,^[^ ^3^ ^]^ centrifugal separation,^[^ ^4^ ^]^ and hydrocyclones.^[^ ^5^, ^6^ ^]^ All these techniques require time and a large amount of energy. For this reason, in the last few years, many studies have focused on developing different membranes to enhance the mechanical filtration of water‐in‐oil and oil‐in‐water emulsions.^[^ ^7^, ^8^, ^9^, ^10^, ^11^, ^12^, ^13^, ^14^ ^]^ Among these, nanofibrous membranes fabricated by electrospinning represent an exciting alternative due to their high flexibility and high separation performance.^[^ ^15^, ^16^, ^17^ ^]^ Many different polymers have been employed to prepare these membranes for oil‐water separation, such as polysulfonamide.^[^ ^18^ ^]^ polyimide,^[^ ^19^ ^]^ polystyrene,^[^ ^20^ ^]^ polyvinylidene fluoride,^[^ ^21^ ^]^ and polyurethane.^[^ ^22^ ^]^

In response to the increasing demand for sustainable and cost‐effective methods for producing fibrous membranes, several studies have examined the use of waste polymers to achieve oil‐water separation. For example, Liu et al. designed an electrospun membrane to separate oil‐water mixtures and emulsions by coating a stainless mesh using waste cigarette filters as raw material.^[^ ^23^ ^]^ Waste polystyrene (PS) was also used by Sow et al. to fabricate superoleophilic fiber‐coated membranes for oil recovery via blow spinning, which showed a separation efficiency of up to 97%.^[^ ^24^ ^]^

Polyethylene terephthalate (PET) is a low‐cost, thermoplastic polyester. This polymer is widely used in packaging, automotive, electrical, electronic, and, particularly, in the textile industry, representing the primary PET market. The increase in PET use, mainly to produce bottles, has made the end‐of‐life management of this material a more crucial issue. Over the years, many different solutions have been developed to recycle PET. Unfortunately, recycled PET (r‐PET) bottles are usually reused to fabricate low‐cost products, so that its market profits are relatively low. Using r‐PET as a basis to produce fibrous membranes for oil‐water separation could result in a reduction in the cost of the raw materials and provide environmental benefits. The increase in the added value of r‐PET products encourages the collection and recycling of this polymer, reducing the amount of plastic released in the environment. Some studies have already been conducted on the use of r‐PET in fibrous filters. Strain et al. reported that the electrospun r‐PET fibrous membrane can be used for smoke filtration.^[^ ^25^ ^]^ Zander et al. successfully developed r‐PET nanofibers to filter particles with sizes of 30 to 2000 nm dispersed in water.^[^ ^26^ ^]^ In a previous work, the oil‐water separation performance of functionalized r‐PET has also been tested and reached separation efficiencies above 98.5%.^[^ ^27^ ^]^

Recently, smart nanofibrous membranes were fabricated to control the oil‐water separation process. Smart materials, which can respond to temperature,^[^ ^28^ ^]^ pH,^[^ ^29^ ^]^ light,^[^ ^30^, ^31^ ^]^ ions,^[^ ^32^ ^]^ electric fields,^[^ ^12^ ^]^ and prewetting, are considered emerging candidates for on‐demand oil‐water separation. Prewetting is the most promising approach because of its facile fabrication and operation.^[^ ^8^, ^11^ ^]^ The membrane used for the prewetting process should exhibit amphiphilic properties, with underwater superoleophobic and underoil superhydrophobic behavior. When this membrane is prewetted with oil, it allows for only oil to pass through while inhibiting water penetration. In contrast, when the membrane is wetted by water, it will enable water to pass through, while the oil remains above the membrane.

According to previous studies, r‐PET nanofibrous membranes were hydrophobic and oleophilic.^[^ ^27^ ^]^ Therefore, a modification is required to obtain an amphiphilic membrane from r‐PET. In this study, a simple and sustainable approach was developed to fabricate amphiphilic membranes from r‐PET and chitosan. The r‐PET membrane was modified with chitosan, a hydrophilic and biodegradable polymer, produced by deacetylation of chitin, one of the most abundant biopolymers extracted by different species of fungi, crustaceans, and insects.^[^ ^33^ ^]^ The deacetylation‐generated protonation of amino groups ensures the solubility of this polysaccharide in dilute acidic aqueous solutions,^[^ ^34^ ^]^ as well as to conferring some functional properties^[^ ^35^ ^]^ and promoting spinnability. The nanofibrous membrane fabricated from the blend of r‐PET and chitosan is expected to have amphiphilic properties due to the presence of the hydrophobic groups of r‐PET and the hydrophilic groups of chitosan. By prewetting the membrane with oil or water, the oil‐water separation could be controlled.

## 2 Results and Discussion

### 2.1 Morphology

**Figure 1:**
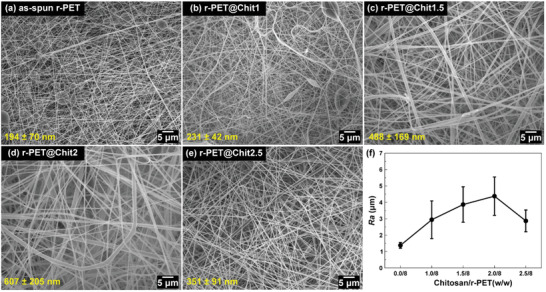
The SEM images of a) as‐spun r‐PET, b) r‐PET@Chit1, c) r‐PET@Chit1.5, d) r‐PET@Chit2, e) r‐PET@Chit2.5; which shows an increase in the diameter of the fibers with a higher concentration of chitosan in solution. f) The plots of chitosan concentration versus roughness for the membrane show a similar trend.

Previous studies^[^ ^36^ ^]^ demonstrated that the morphology of membranes significantly affects their surface properties, such as wettability and oil‐water separation performance. The fiber diameter and the membrane roughness were measured using a field‐emission scanning electron microscope (FE‐SEM) and laser microscopy. **Figure** **1** shows the morphology of the fibers electrospun from the polymeric solutions containing different amounts of chitosan. Smooth and beaded fibers were produced from the polymeric solution containing r‐PET only; however, the roughness and the mean diameter of the fibers were relatively low (1.4 ± 0.2 µm and 194 ± 70 nm, respectively). This kind of fiber structure is formed due to the low viscosity of the polymeric solution under the given applied voltage.^[^ ^37^ ^]^

Adding chitosan to the polymeric solution, the shape of the fibers was more homogeneous, and the beads completely disappeared. In addition, as expected,^[^ ^38^, ^39^ ^]^ the fiber diameter generally increased with the increase in the chitosan content and reached 607 ± 205 nm when the chitosan/r‐PET ratio was 2.0/8. A singular trend in the fiber diameters was observed for the membranes with an initial concentration of chitosan in solution equal to 2.5wt% (r‐PET@Chit2.5), which showed a sudden decrease in the diameter. To better understand this phenomenon, it is necessary to consider the polymeric solution conductivity and viscosity, which are the two types of properties of the polymeric solutions with the greatest effect on the fiber dimension during electrospinning.^[^ ^40^, ^41^ ^]^ As shown in Figure S1, Supporting Information, the polymeric solution conductivity increased linearly from 1.09 to 35.8 µS cm^−1^ with increasing chitosan content. The viscosity of the polymeric solution also showed a gradual increase with the chitosan content. This experimental evidence showed that for low concentrations, the increase in viscosity strongly affected the electrospinning process,^[^ ^25^, ^37^ ^]^ while for polymer concentrations higher than 2 wt%, the conductivity was the dominant parameter. Therefore, the decrease in the diameter of the prepared fibers of r‐PET@Chit2.5 could be explained.

The roughness of the membranes was investigated using noncontact laser profilometry, as shown in Figure 1f and Figure S2, Supporting Information. The analysis revealed a close correlation between the membrane roughness and the dimension of the fibers. The results showed that the *R*_a_ of the as‐spun r‐PET membrane was relatively small (1.4 ± 0.2 µm) compared with that of r‐PET@Chit2 (4.4 ± 1.5 µm). Furthermore, similar to the mean diameters, the roughness of r‐PET@Chit2.5 was found to be lower than that of r‐PET@Chit2.

### 2.2 Chemical Composition

**Figure 2:**
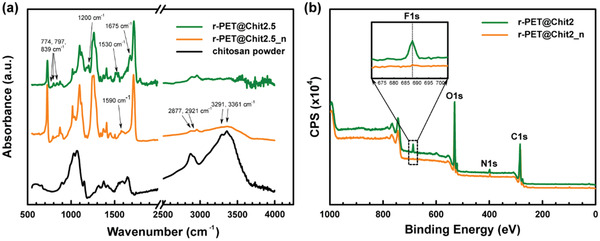
a) From the FT‐IR spectra of chitosan powder, r‐PET@Chit2.5, and r‐PET@Chit2.5 after the neutralization (r‐PET@Chit2.5), the presence of a residual TFA salt is evident from the characteristic peaks at 1675, 1530, 1200, 893, 797, and 774 cm^−1^. After the process of neutralization, the solvent salt appears eliminated, while the presence of chitosan on the surface of the fibers seems to be unaffected by the chemical treatment. b) The XPS wide‐scan spectra of r‐PET@Chit2.5 before and after the neutralization confirmed the elimination of the traces of the residual salt on the surface of the fibers by the neutralization process.

The chemical composition of the fibers can play a crucial role in determining the surface properties of the membranes and affect their wettability and filtration performance. For this reason, the IR spectra of all membranes were measured by a Fourier‐transform infrared spectroscopy equipped with a universal attenuated total reflectance accessory (ATR‐FT/IR). As shown in **Figure** **2**a and Figure S3, Supporting Information, the analyzed sample spectra contained absorption peaks at 1675 and 1530 cm^−1^ attributed to protonated amino (NH_3_^+^) stretching. In addition, the weak peaks at 3300 and 3400 cm^−1^ were attributed to the amino (NH_2_) stretching. The absorption peaks at 1200 cm^−1^ and the broad peak at ≈840–720 cm^−1^ indicated the presence of carboxylate (COO^−^) and amino trifluoroacetate (CF_3_COO^−^ NH_3_^+^) in the fibers, respectively.^[^ ^42^, ^43^, ^44^ ^]^

Figure 2b shows the X‐ray photoelectron spectroscopy (XPS) wide‐scan spectra of r‐PET@Chit2.5 before and after the neutralization process. The XPS spectrum of r‐PET@Chit2.5 before the neutralization process contained peaks at 285, 532, 399, and 688 eV corresponding to the binding energies of C1s, O1s, N1s, and F1s, respectively. The signal centered at 688 eV originated from the C—F binding energy distributed on the surface of the membrane before the neutralization process.

As discussed further below, some residual traces of trifluoroacetic acid (TFA) salt were detected on the surface of the fibers from the chemical analysis. These results suggest that when the membrane contacts water, the salt residues in the membrane can dissolve and contaminate the liquid with toxic components and reduce its pH (which can lead to further chitosan dissolution). Therefore, it is considered necessary to neutralize the membrane and remove the TFA traces. For this reason, the neutralization process previously mentioned should be performed with all the membranes and, more notably, did not affect the morphology of the fibers, as confirmed by FE‐SEM images (Figure S4, Supporting Information). On the other hand, the chemical treatment affected the roughness of the membrane by homogeneously flattening the fibrous mats (Figure S4, Supporting Information).

As shown in Figure 2, the IR spectrum of the neutralized r‐PET@Chit2.5 did not contain the peaks attributed to the TFA salts or the peaks at 3400, 3300, 2921, 2877, and 1590 cm^−1^ corresponding to N—H and O—H stretching (first two signals), C—H symmetric and asymmetric stretching (second two signals), and N—H bending (last signal).^[^ ^45^, ^46^ ^]^ Furthermore, the XPS data (Figure 2b) confirmed the results, revealing that the residual fluorine did not exceed 0.3 at%, as shown in Table S1, Supporting Information.

The amount of chitosan on the surface of the fibers is crucially important to achieve the desired wettability and filtration properties. Therefore, the XPS measurements of the neutralized membranes were also useful to investigate the chemical composition of the fiber surface. Here, the concentrations of atomic compositions of pure chitosan powder and r‐PET membrane were measured to estimate the atomic compositions in the neutralized r‐PET@Chit membranes at the fiber surface (**Table** **1**). An estimation of the N composition is the best approach to assess the chitosan concentration at the fiber surface. The results revealed that the amount of chitosan in the neutralized r‐PET@Chit membranes was higher than the value estimated from the bulk, which could be caused by the high concentration of chitosan dispersed on the fiber surface. Similarly, other elements and energy‐dispersive X‐ray spectroscopy (EDX) element mappings were also evaluated, and the results are summarized in Figure S5, Supporting Information.

**Table 1:** Comparison between the atomic percentage of nitrogen on the neutralized membrane surface and the calculated results

Sample	%at N	
	Membrane	Theoretical
r‐PET@Chit1_n	1.21	0.67
r‐PET@Chit1.5_n	1.53	0.97
r‐PET@Chit2_n	1.95	1.23
r‐PET@Chit2.5_n	1.92	1.49

### 2.3 Wettability

**Figure 3:**
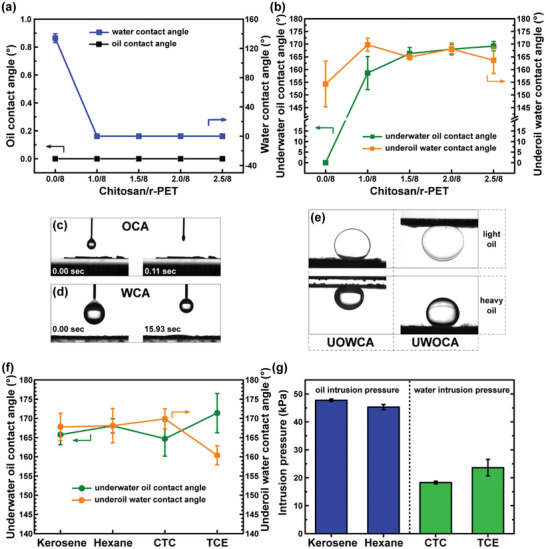
The amphiphilic property of the designed membranes was confirmed by the a) oil (hexane) and water CA analysis. The b) underwater oil and underoil water contact angles showed the underoil superhydrophobicity and the underwater superoleophobicity of the membranes with different contents of chitosan. Images of the OCA (c) and WCA (d) before and after a drop of liquid was completely absorbed by the r‐PET@Chit2_n membrane. e) UWOCA and UOWCA of a light (hexane) and heavy (TCE) oil for r‐PET@Chit2_n. f) Underwater oil and underoil water contact angles for r‐PET@Chit2_n using different kinds of oils confirmed the behavior just observed also using kerosene, carbon tetrachloride (CTC), and tetrachloroethylene (TCE). g) The r‐PET@Chit2_n membrane was also used to measure the oil and the water intrusion pressure for the same oils as before.

The wettability of the membrane was investigated by measuring the contact angle, as shown in **Figure** **3**. The effect of the chitosan concentration in the fibers on the water contact angle and oil contact angle was studied. The as‐spun r‐PET membrane showed high water contact angle (WCA) values of 134.0°, which was due to the high surface roughness, as already observed in a previous work.^[^ ^27^ ^]^ In contrast, by adding chitosan to the fibers, the membrane showed superhydrophilic behavior without affecting the already observed superoleophilicity (Figure 3a). The modified membrane wettability was attributed to the presence of the biopolymer on the surface of the fibers. Chitosan provides more hydrophilic groups for interaction with water.^[^ ^47^ ^]^ The amphiphilic properties of the membrane were further investigated by immersing the mats in water and hexane and measuring the contact angle against oil and water, respectively. This approach results in the formation of a stable solid/water/oil three‐phase system by prewetting the membranes,^[^ ^10^ ^]^ resulting in underwater superoleophobicity and underoil superhydrophobicity (Figure 3b). Except for the case of the as‐spun r‐PET membrane in water, when the sample was immersed in a liquid (water or oil), the respective liquid formed a layer around the fibers. When another immiscible liquid was brought into contact with the surface of the membrane, it was repulsed by the layer of water/oil and formed a quasi‐spherical shape.^[^ ^7^, ^10^, ^11^ ^]^ The presence of chitosan allowed for the membrane to repel different solvent categories by prewetting it with the opposite liquid. As observed experimentally, the underwater oil contact angle (UWOCA) and underoil water contact angle (UOWCA) did not change much as a function of the chitosan content and exceeded 150° for each sample. In particular, the r‐PET@Chit2_n mat showed the highest values, a UWOCA of 168.0 ± 1.9° and a UOWCA of 168.1 ± 2.5°. The modification of the wettability properties as a response to different solvent types was also examined by repeating the contact angle analysis with kerosene, carbon tetrachloride (CTC, CCl_4_), and tetrachloroethylene (TCE, C_2_Cl_4_). As shown in Figure 3f, the UWOCA and the UOWCA did not change significantly, resulting in contact angles between 160° and 175° for all the oils tested.

Furthermore, the maximum height of the column of liquid that the membrane could retain before the first drop passed through was measured by evaluating the intrusion pressure for the different oils. In the case of amphiphilic membranes, it is necessary to measure both water and oil intrusion pressure. When the membrane is wetted by a liquid (e.g., oil) with a good affinity (CA ≈ 0°), it attaches to the surface of the fibers. If a drop of another liquid (e.g., water) is deposited on this system, the surface tension at the interface between the liquid‐liquid‐air phases creates an equilibrium of forces (Figure S6, Supporting Information), which allows the drop to rest on the membrane. The pressure that needs to be applied by the deposited liquid to the pore to overcome the surface tension is the intrusion pressure, which can be calculated using the Young‐Laplace theory.^[^ ^48^, ^49^ ^]^

(1) $$\begin{array}{c} \Delta P=\frac{2\gamma_{{}_{L}}}{r}=-\frac{2\gamma_{{}_{L}}\cos\theta }{R} \end{array}$$

where Δ*P* is the liquid intrusion pressure, γ_L_ is the surface tension of the deposited liquid, *r* is the curvature radius of the meniscus, θ is the contact angle of the liquid on the surface of the fibers, and *R* is the equivalent pore radius of the membrane. For these measurements, only r‐PET@Chit2_n was used because it showed the most promising values of UWOCA and UOWCA.

The water intrusion pressure was measured by prewetting the membrane with heavy oil, and the oil intrusion pressure was measured using light oils on water‐prewetted membranes. In fact, in a practical case, the heavier liquid reached the filter prior to the lighter liquid and passed through the membrane, so that the other fluid accumulated above the membrane surface. The amount of liquid that the membrane can sustain before its filtration capacity is compromised and is therefore a crucial parameter. As shown in Figure 3g, the oil intrusion pressure was almost constant for the two different solvents used (47.7 ± 0.5 kPa for kerosene and 45.3 ± 1.0 kPa for hexane). On the other hand, the water intrusion pressure was lower, and the value changed from 18.3 ± 0.5 kPa for carbon tetrachloride to 23.6 ± 3.0 kPa for tetrachloroethylene.

In addition, the maximum stress and elongation at break of r‐PET@Chit2_n were measured because an evaluation of the mechanical stability is very important for any practical application. As a result, as shown in Figure S7, Supporting Information, the calculated maximum stress and elongation at break were 2.2 ± 0.2 MPa and 9.6 ± 1.1%, respectively.

### 2.4 Separation Performance

**Figure 4:**
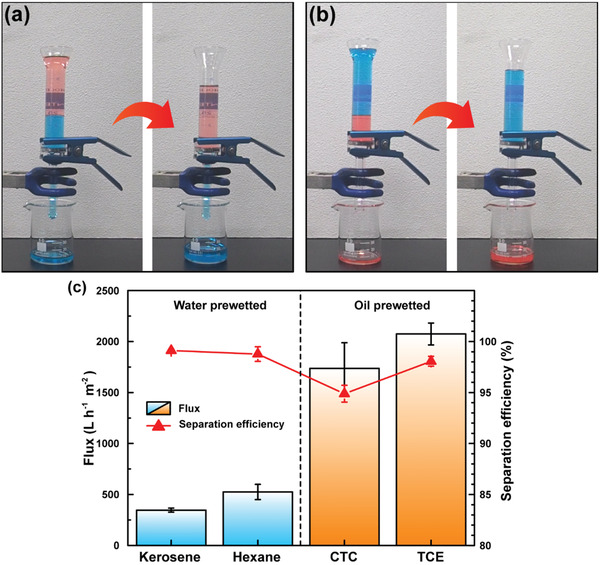
The apparatus used to measure the mixture separation in case of a) light and b) heavy oils. Water and oil were colored by a blue dye and red dye, respectively. c) Flux and separation efficiency measured using the r‐PET@Chit2_n membrane for different kinds of water/oil mixtures.

**Figure 5:**
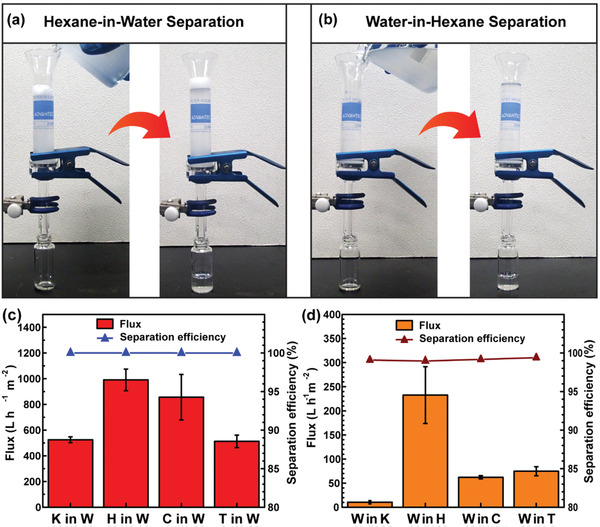
The apparatus used for the emulsion separation test of the r‐PET@Chit2_n membrane in case of a) oil‐in‐water and b) water‐in‐oil emulsions. c) Separation efficiencies and fluxes for the oil‐in‐water emulsions for different types of oils: kerosene (K), hexane (H), carbon tetrachloride (C), and tetrachloroethylene (T). d) Separation efficiencies and fluxes for the water‐in‐oil emulsions using the same types of solvents as before.

The contact angles and antifouling properties of the designed membrane showed promising results regarding its oil‐water separation. For this reason, filtration experiments were performed with the r‐PET@Chit2_n membrane using different types of oil/water mixtures and emulsions. In particular, the solvents used were kerosene, hexane, CTC, and TCE. The setup used in this study is shown in **Figures** **4**a,b and **5**a,b; Videos S1 and S2, Supporting Information. The separation mechanism could be explained from estimating the intrusion pressure (Δ*P*) by Equation (1).^[^ ^27^ ^]^ It was found that when oil or water was brought in contact with the membrane in air, both had a CA close to 0°. This corresponds to Δ*P* < 0, suggesting that the liquid flows freely through the membrane. On the other hand, if the membrane was prewetted by oil (or water) and some water (or oil) was deposited on its surface, θ > 90° indicated a positive value of the intrusion pressure. In this kind of system, the liquid is contained in the membrane. This mechanism, achieved by the designed membrane, results in a controllable separation of water from oil or vice versa by prewetting the membrane with the corresponding liquid.

A mixture filtration test was performed by filtering 20 mL of the oil/water mixture with a volume ratio of 1:1 with the prewetted membranes. The results showed a relatively high efficiency for the mixture separation: 94.9% for CTC, 98.1% for TCE, 98.8% for hexane, and 99.1% for kerosene mixtures (Figure 4c). The measured fluxes were between 346.2 and 524.5 L h^−1^ m^−2^ for the light oil‐water mixture, 1737.1 and 2073.8 L h^−1^ m^−2^ for the heavy oil‐water mixtures (Figure 4c). Compared with other sustainable amphiphilic membranes for oil‐water mixture separation (Table S2, Supporting Information), the fluxes were higher, although the efficiencies remained competitive. The large difference between the oil and water fluxes is probably due to the difference in the viscosity of the liquids. As shown in Table S3, Supporting Information, water has a higher viscosity than the heavy oils. In addition, the heavy oil/water mixtures had a higher density than the other two mixtures, so that in the first case, the liquid is pushed more easily into the pores.

**Figure 6:**
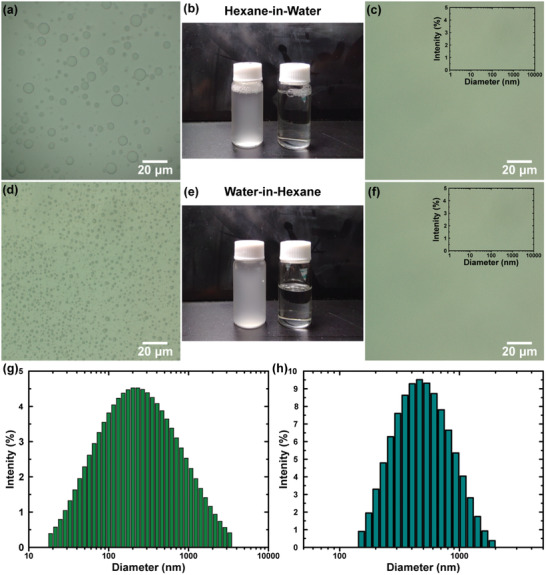
a–f) Optical microscope images of the hexane in water and water in hexane emulsions before and after the filtration. In (c) and (f) are also reported the droplet size distribution of hexane (H) in water (W) and water (W) in hexane (H) emulsions after the separation. g,h) The droplet size distribution of the two emulsions before the filtration.

On the other hand, the surfactant‐stabilized emulsions were stirred, and using dynamic light scattering (DLS), the dimensions of the dispersed droplets were measured. As shown by the digital images in **Figure** **6** and Figure S8, Supporting Information, the initial emulsions appeared milky, while after filtration, it seemed that the membrane successfully separated the dispersed phase from the dispersant. The optical microscope images (Figure 6 and Figure S8, Supporting Information) also confirmed the presence of small droplets in the feed and an apparent absence of small droplets in the filtrated liquid. In addition, no signals were observed in the DLS measurements, which could indicate that the concentration of the dispersed phase was below the detection limit of the instrument. The oil concentration in water and the water concentration in oil, collected by gas chromatography and Karl Fisher titration, respectively, were lower than 104 ppm. The separation efficiencies calculated by Equation (4) were found to be > 99% (Figure 5c,d). The flux values were between 512 and 991 L h^−1^ m^−2^ for the oil in water emulsions and 10 and 233 L h^−1^ m^−2^ for the water‐in‐oil emulsions (Figure 5c,d). The fluctuation in the oil emulsion fluxes could be attributed to the difference in the liquid viscosity. The viscosity of hexane is almost six‐fold lower than that of kerosene (Table S3, Supporting Information). With respect to other membranes for on‐demand oil‐water emulsion separation (Table S4, Supporting Information), the filter with our design had relatively low fluxes, while the efficiency was among the highest. Consequently, the main advantage of the membrane in this study appears to be its potential as a sustainable new material, which can be applied for both mixture and emulsion separation.

## 3 Conclusions

In summary, low‐cost and environmentally friendly membranes for controllable oil‐water separation by prewetting were successfully fabricated. Therefore, the filter can be considered suitable to meet the growing demand for low environmental impact solutions for the separation of oil and water in wastewater purification treatments and ocean cleaning.

This study revealed the possibility of controlling the morphology, chemical composition, and wettability of the membranes through chitosan concentration in the spun polymeric solutions. Besides, the great versatility of the object of this study has been demonstrated since, through a simple prewetting process, it is possible to control the type of filtered liquid. In fact, thanks to the amphiphilic properties of the membrane, we obtain a product that can be easily used to purify oil from water or vice versa. Finally, the resulting filter has shown antifouling properties and separation efficiency equal or superior to many other examples in the literature. Moreover, convincing performances have been observed both in the separation of mixtures and oil and water emulsions, thus confirming this solution's remarkable versatility.

## 4 Experimental Section

##### Materials

Recycled PET pellets from postconsumer PET water bottles (CR‐8816) were kindly provided by Dr. Kazushi Yamada (Advanced Fibro‐Science, Kyoto Institute of Technology, Kyoto, Japan). Chitosan and sodium dodecylbenzenesulfonate were purchased from Sigma‐Aldrich (75–85% deacetylated, low molecular weight: 50–190 kDa). TFA, carbon tetrachloride, tetrachloroethylene, and sorbitan monooleate were obtained from Wako Co., Osaka, Japan. Kerosene, hexane, and sodium hydrogen carbonite were provided by Nacalai Tesque, Kyoto, Japan. All chemicals were used as received without further purification.

##### Electrospinning and Neutralization

The first step for the electrospinning process was the preparation of five polymeric solutions with a constant amount of r‐PET and different contents of chitosan. The reference polymeric solution contained 8 wt% r‐PET. The r‐PET solution was prepared by adding polymeric pellets to TFA and mixing with a planetary centrifugal mixer (ARE‐310, Thinky Co., Tokyo, Japan) at 2000 rpm for 28.5 min, followed by degassing at 2200 rpm for 1.5 min. The other polymeric solutions contained an increasing content of chitosan from 1 to 2.5 wt%. In this case, the two polymers were added to the polymeric solution separately for better mixing. First, chitosan was added to TFA and mixed according to the above procedure. Then, the r‐PET pellets were added, and the as‐prepared polymeric solution was mixed again. After this process, the compounds were magnetically stirred at room temperature (RT) for ≈10 h to obtain a homogeneous polymeric solution.

Fibrous membranes were prepared using a home‐built electrospinning machine. The discharge volume of the polymeric solution during electrospinning was kept at 0.5 mL h^−1^ by a syringe pump (KDS‐100, KD Scientific Inc., Massachusetts, USA). A high voltage power supplier (HVU‐30P100, MECC Co., Japan) was used to generate a high voltage of 15 kV at the tip of a 30 G needle. A rotating drum with a diameter of 61 mm covered with a nylon mesh (No. 34) was used as a collector and fixed at 12.5 cm from the needle at a constant rotation of 120 rpm. The temperature and relative humidity during electrospinning were monitored with a hygrothermograph and maintained at 22.5 ± 4.0 °C and 42 ± 8%, respectively. Four membranes were obtained using the above procedure with different chitosan/r‐PET ratios in the respective fibers: 0.0/8, 1.0/8, 1.5/8, 2.0/8, and 2.5/8. The membranes are denoted as‐spun r‐PET, r‐PET@Chit1, r‐PET@Chit1.5, r‐PET@Chit2, and r‐PET@Chit2.5.

After the electrospinning process, the membranes were neutralized by immersing in a supersaturated water solution of sodium hydrogen carbonate (NaHCO_3_) for at least 6 h at room temperature.^[^ ^44^ ^]^ In particular, the supersaturated solution was prepared by dissolving ≈95.5 g L^−1^ of NaHCO3 in water and filtering the solution after a magnetically stirring to eliminate the excess. Then, the membranes were washed with distilled water several times and dried at 50 °C for 4 h. The membranes obtained after the neutralization process were denoted as r‐PET@Chit1_n, r‐PET@Chit1.5_n, r‐PET@Chit2_n, and r‐PET@Chit2.5_n.

##### Characterization

Membrane morphology and chemical composition maps were obtained by field‐emission scanning electron microscopy (FE‐SEM) (JEOL‐7600, JEOL Ltd., Japan), equipped with an Oxford energy dispersive X‐ray spectrometer. A platinum layer of 30 nm was sputtered on the sample surfaces prior to collecting images using an electron beam accelerated to 15 kV. Diameters were measured by processing 100 fibers of each membrane with image processing software (ImageJ). A 3D laser scanning microscope (VK‐2000, Keyence Co., Japan) was used to determine surface roughness via noncontact laser profilometry. The viscosity of the solutions was evaluated using a vibro viscosimeter (SV‐1 and SV‐100, A&D, Tokyo, Japan). The conductivity was measured by an Oakton PC700 pH/mV/Conductivity/°C bench meter (Oakton Instruments, Vernon Hills, IL, USA). An FT/IR spectrometer (FT/IR 4700, JASCO International Co., Japan) was used to record infrared spectra of the samples in the range between 4000 and 400 cm^−1^. The chemical composition of the membrane surface was investigated using XPS (JEOL 9010, JEOL Ltd., Japan). The mechanical properties of the neutralized membranes were evaluated using a universal tensile testing machine (TENSILON RTF‐1210, A&D Co., Japan) with a crosshead speed of 1 mm s^−1^ and a load cell of 100 *N*. The samples were cut in dog bone‐shaped specimens (type 5B in BS ISO 527: 2012) and then attached to a tensile test stand fabricated from a paper window frame of 20 × 30 mm with a window size of 4 × 25 mm. Contact angle (CA) analysis was conducted using a Phoenix 300 contact angle system (Kromtek Co., Malaysia) at RT. The results were further evaluated using image processing software (ImageJ). The membranes were attached to a glass slide and placed in air, water, or oil to measure the WCA, OCA, UWOCA, and UOWCA. During the measurements, four different types of oils were used: kerosene, hexane, CTC, and TCE. To evaluate the amount of liquid that the membrane could retain without losses, the intrusion pressure was measured using a homemade testing system described in the previous study.^[^ ^27^ ^]^

##### Oil‐Water Separation Tests

The ability of the membrane to infiltrate both oil‐water mixtures and emulsions was evaluated using a dead‐end filtration apparatus. The r‐PET@Chit2_n membrane was cut into circles with diameters of 25 mm and fixed between a filter holder, KGS‐25 (Advantec Co., Japan), with a stainless‐steel supporter.

In the mixture separation, four different oil types were used to conduct the test: kerosene, hexane, CTC, and TCE. The water was colored with a blue dye, and the oil was colored with a red dye. The membranes were prewetted by oil or water, and 20 mL of oil/water mixture (volume ratio, 1/1) was poured into the filter holder. When separating water from the oil/water mixture, the membranes were prewetted with water, whereas when separating oil, the membranes were prewetted with oil. During the test, the heaviest liquid, filtered by the membrane, was collected in a conical beaker. The flux (*J*) through the membrane was calculated using the equation:

(2) $$\begin{array}{c} J\;=\frac{V}{At} \end{array}$$

where *V* is the volume of the collected liquid, *t* is the time of collection and *A* is the effective area of filtration. The filtration efficiency was calculated using the following equation:

(3) $$\begin{array}{c} \eta_{m}=\frac{M}{M_{0}}\;\times 100\% \end{array}$$

where η_m_ is the separation efficiency, *M* is the weight of the liquid collected in the beaker, and *M*_0_ is the initial weight before the separation.

The emulsions were prepared using two types of surfactants: sorbitan monooleate for the water‐in‐oil emulsions and sodium dodecylbenzenesulfonate for the oil‐in‐water emulsions. First, the continuous phase was weighed, and 0.1 wt% of surfactant was added. The prepared solution was then mixed with an Ultra Turrax IKA T‐18 disperser (IKA; Werke GmbH & Co., KG, Staufen, Germany) for 5 min at 18 000 rpm to homogenize the mixture. Then, the dispersed phase was added at a concentration of 1 wt% and mixed again for 5 min. The size of the emulsified particles was measured by a dynamic light scattering (DLS) analyzer (ELSZ‐1000, Otsuka Electronics Co., Ltd., Osaka, Japan).

The specimens were prepared in the filter holder, which was then filled with 15 mL of emulsion and kept constant to avoid modifying the pressure applied to the membrane. After 1 min, the filtrated liquid was weighed to calculate the flux using Equation (2). The concentration of the dispersed phase after filtration was measured by gas chromatograph‐mass spectrometer (GCMS‐QP2010 Ultra, Shimadzu, Kyoto, Japan) for the oil‐in‐water emulsions and a Karl Fischer Moisture Titrator MKC‐710 (Kyoto Electronics Manufacturing Co., Kyoto, Japan) for the water‐in‐oil emulsions. The results of these measurements were then used to calculate the filtration efficiency by using the following equation:

(4) $$\begin{array}{c} \;\eta_{e}=\frac{C_{i}-C_{f}}{C_{i}}\;\times 100\% \end{array}$$

where η_e_ is the filtration efficiency, and *C*_i_ and *C*_f_ are the emulsion concentrations before and after filtration, respectively.

## Conflict of Interest

The authors declare no conflict of interest.

## Supporting information

Supporting Information

Supplemental Video 1

Supplemental Video 2
