# Supplementary material for: Chitosan‐Functionalized Recycled Polyethylene Terephthalate Nanofibrous Membrane for Sustainable On‐Demand Oil‐Water Separation
Source: Glob Chall. 2021 Jan 12;5(4):2000107. doi: 10.1002/gch2.202000107 (PMC8025399; doi:10.1002/gch2.202000107)
Supplement: Supplementary file 1 — Supporting Information [file GCH2-5-2000107-s001.pdf]

© 2021 Wiley-VCH GmbH

Supporting Information

for *Global Challenges,* DOI: 10.1002/gch2.202000107

Chitosan-Functionalized Recycled Polyethylene Terephthalate Nanofibrous Membrane for Sustainable On-Demand Oil-Water Separation

Andrea Baggio, Hoan N. Doan, Phu P. Vo, Kenji Kinashi, Wataru Sakai, Naoto Tsutsumi, Yasuro Fuse, and Marco Sangermano*

© Copyright 2020. WILEY-VCH GmbH.

Supporting Information

Chitosan-functionalized Recycled Polyethylene Terephthalate Nanofibrous Membrane for Sustainable On-Demand Oil-Water Separation

Andrea Baggio, Hoan N. Doan, Phu P. Vo, Kenji Kinashi, * *Wataru Sakai, Naoto Tsutsumi, Yasuro Fuse, and Marco Sangermano*

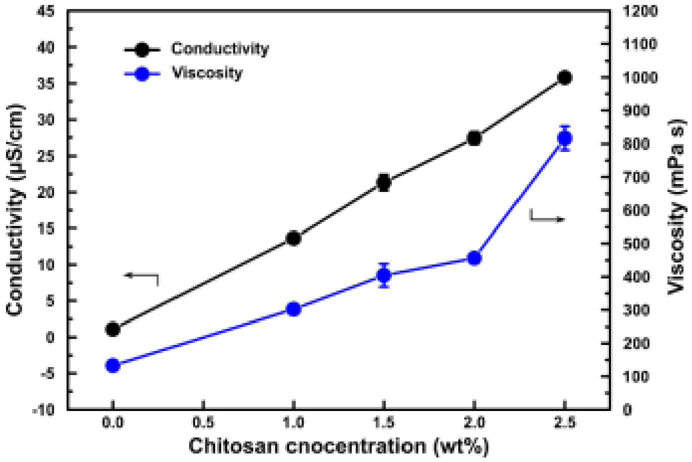

Figure S1. The viscosity and conductivity of the solutions containing different chitosan concentrations were used to explain the results measured for the fibers' average diameter.

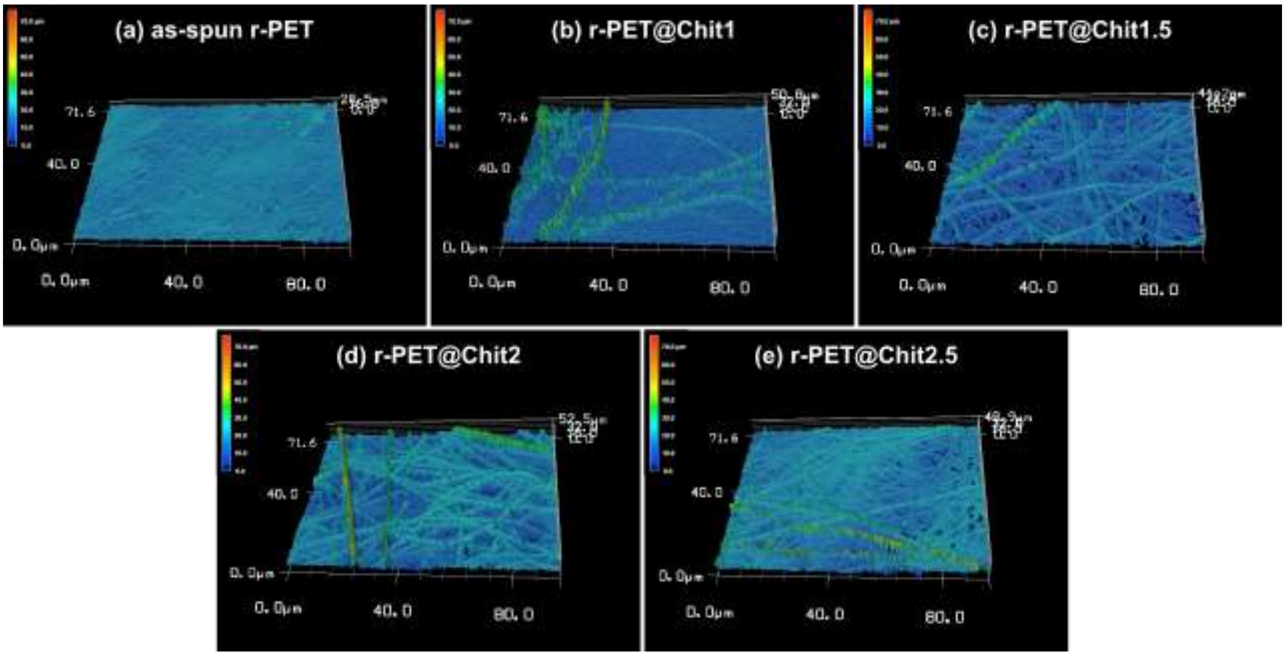

Figure S2. Optical profilometry images of the membranes for different chitosan concentrations help visualize the measured roughness of the membranes.

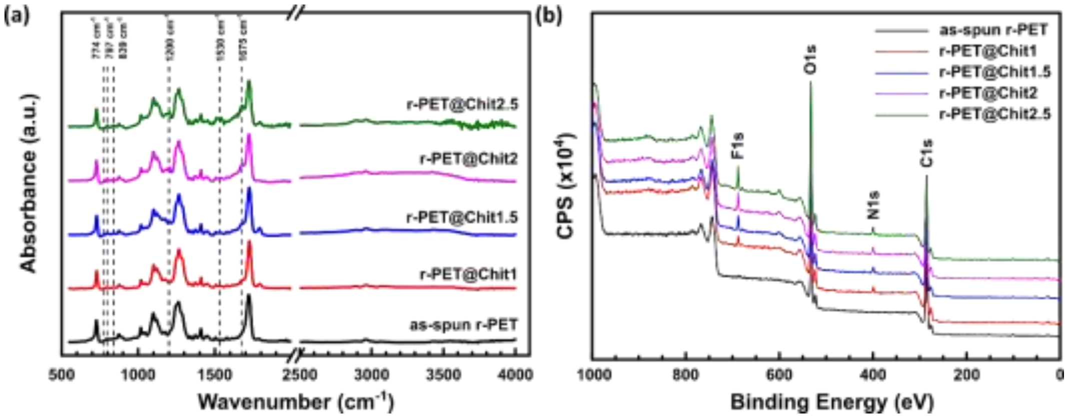

Figure S3. (a) FT-IR and (b) XPS spectra of the membrane with different concentrations of chitosan. The FT-IR peaks related to the presence of the TFA salt are indicated, as well as the elements detected by the XPS analysis.

Table S1. The atomic percentage of elements in membranes before (as-spun) and after neutralization measured by the XPS wide scan.

Sample	at % C		at % N		at% O		at% F	
	as-spun	neutralized	as-spun	neutralized	as-spun	neutralized	as-spun	neutralized
r-PET@Chit1	66.90	69.20	1.75	1.21	30.26	29.31	1.11	0.29
r-PET@Chit1.5	64.77	69.34	2.50	1.53	30.76	29.00	1.99	0.14
r-PET@Chit2	63.91	68.53	2.52	1.95	31.50	29.34	2.09	0.18
r-PET@Chit2.5	62.86	68.92	2.88	1.92	31.34	28.94	2.93	0.24

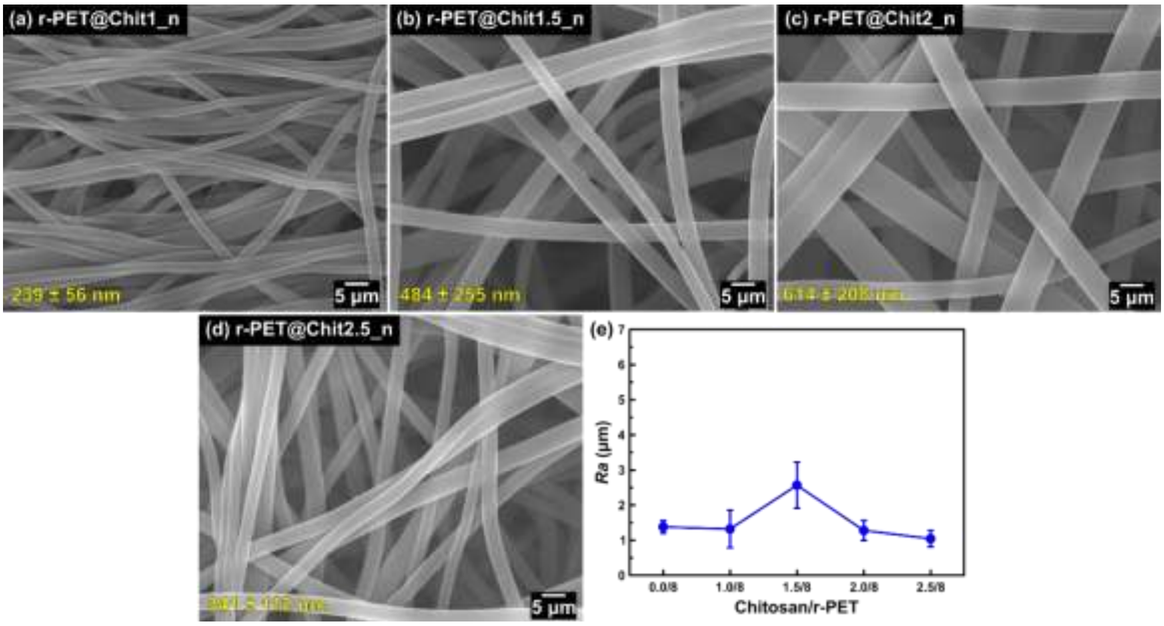

Figure S4. The SEM images of (a) r-PET@Chit1_n, (b) r-PET@Chit1.5_n, (c) r-PET@Chit2_n, (d) r-PET@Chit2.5_n show the irrelevance of the neutralization process on the morphology of the fibers. (e) The values of the roughness of the neutralized membranes evidence homogenization in the surface of the membranes after the chemical treatment.

The atomic percentage of elements calculation

The atomic percentage of the prepared membranes was compared with an expected composition, calculated from the XPS analysis of the pure chitosan powder and the r-PET fibers. In order to reach this purpose, the following equations were used:

$$\%at(N)_{chit} = \frac{n^o\ atoms(N)_{chit}}{n^o\ atoms_{tot_{chit}}} = \frac{n(N)_{chit}}{n_{tot_{chit}}} \cdot \frac{N_A}{N_A} = \%mol(N)_{chit}\ \#(S1)$$

where $\%at(N)_{chit}$ is the atomic percentage of carbon in chitosan powder (measured by the XPS), $n(N)_{chit}$ is the number of moles of carbon in chitosan and $n_{tot_{chit}}$ is the total number of moles in chitosan, N_A is the Avogadro number, and $\%mol(N)_{chit}$ is the molar percentage of carbon in chitosan powder;

$$\%mol(N)_{chit} \cdot AM(N) = \frac{m(N)_{chit}}{n_{tot_{chit}}}\#(S2)$$

where $AM(N)$ is the atomic mass of carbon. Analogous calculations were performed also form the atomic percentage of oxygen and nitrogen in chitosan. After that, the weight percentage of each component was calculated by the following equations:

$$\frac{m(C)_{chit}}{n_{tot_{chit}}} + \frac{m(O)_{chit}}{n_{tot_{chit}}} + \frac{m(N)_{chit}}{n_{tot_{chit}}} = \frac{m_{tot_{chit}}}{n_{tot_{chit}}}\ \#(S3)$$

$$\frac{m(N)_{chit}}{n_{tot_{chit}}} \cdot \left(\frac{m_{tot_{chit}}}{n_{tot_{chit}}}\right)^{-1} = \frac{m(N)_{chit}}{m_{tot_{chit}}} = \%wt(N)_{chit}\ \#(S4)$$

where $m_{tot_{chit}}$ is the total mass of chitosan and $\%wt(C)_{chit}$ is the weight percentage of carbon in chitosan. The same procedure was then used also with the other components of chitosan and with all the elements of r-PET. So, the theoretical homogeneous composition of the fibers was so calculated:

$$\%wt(N)_{chit} \cdot \%wt(chit)_{fiber} + \%wt(N)_{rPET} \cdot \%wt(rPET)_{fiber} = \%wt(N)_{fiber}\ \#(S5)$$

$\%wt(chit)_{fiber}$ and $\%wt(chit)_{fiber}$ – respectively the weight percentage of chitosan and recycled PET in fibers – were known by the amount of material used to prepare the solution. Finally, the atomic composition of the fibers for each component was found:

$$\frac{\%wt(N)_{fiber}}{AM(N)} \cdot \frac{1}{\frac{\%wt(C)_{fiber}}{AM(C)} + \frac{\%wt(O)_{fiber}}{AM(O)} + \frac{\%wt(N)_{fiber}}{AM(N)}} = \%at(N)_{fiber}\ \#(S6)$$

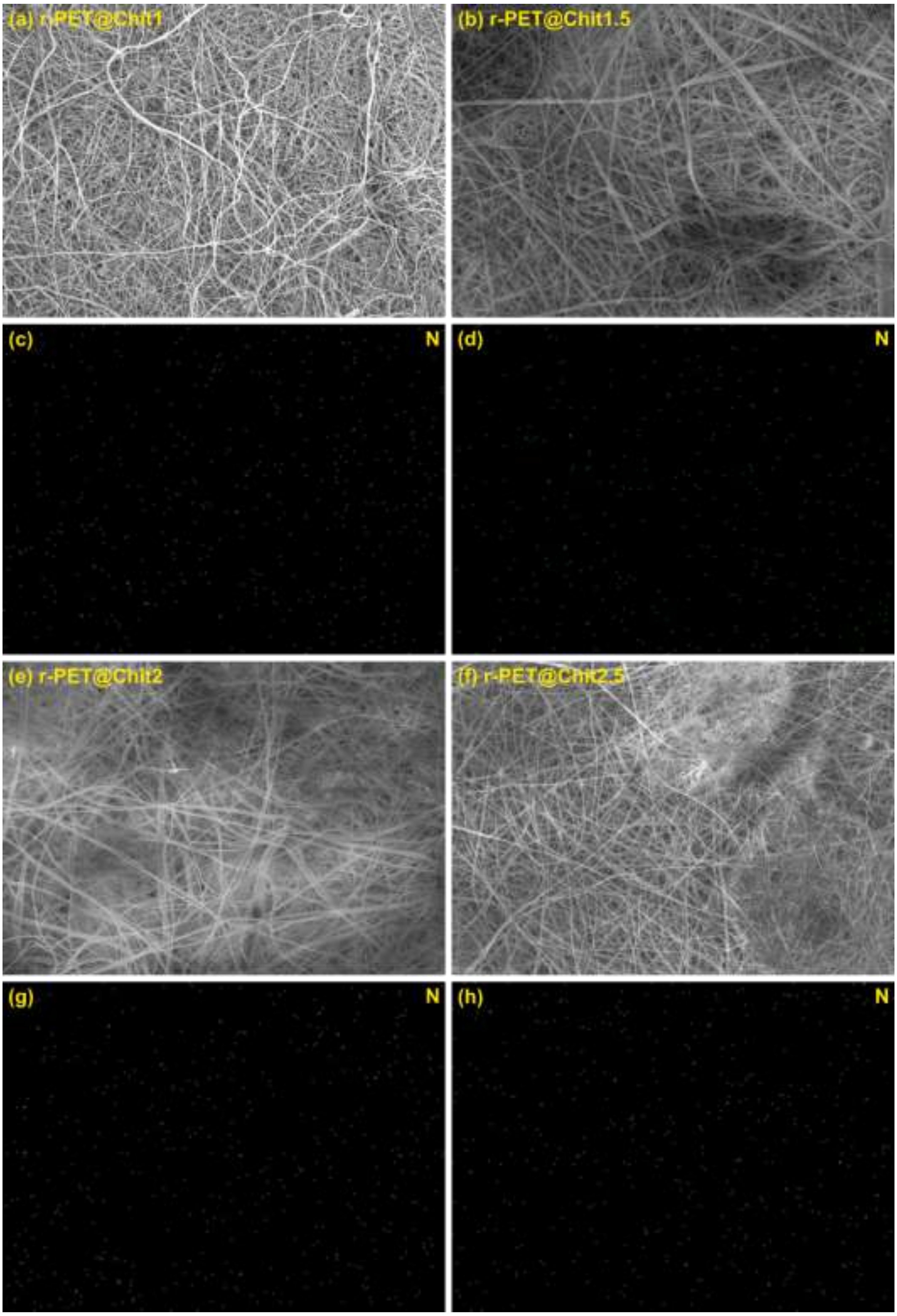

Figure S5. SEM images and elemental mapping of nitrogen detected by EDX for the rPET@Chitosan membranes at different chitosan concentrations: (a) and (c) 1 wt%, (b) and (d) 1.5 wt%, (e) and (g) 2 wt%, (f) and (h) 2.5 wt%. These images show the homogeneous spatial distribution of chitosan by tracing the amino groups through hydrogen atoms.

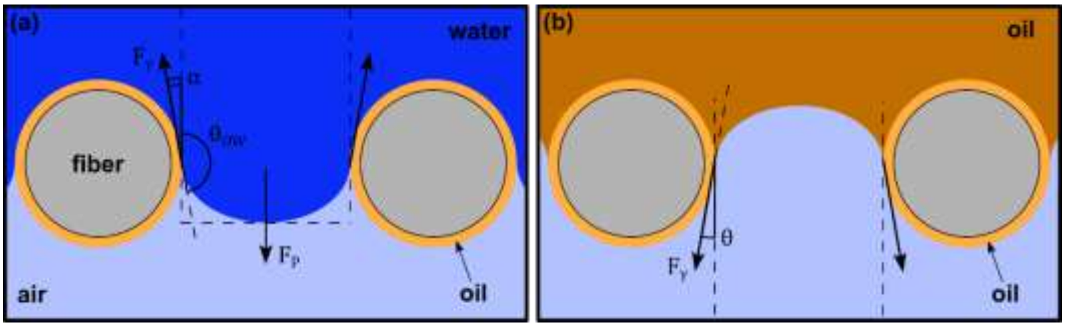

Figure S6. Schematic representation of the filtration mechanism of the membranes prewetted by the oil. When the CA is > 90°, the deposited liquid's surface tension allows it to be retained on the membrane's surface; otherwise, the standing liquid is free to flow through the filter.

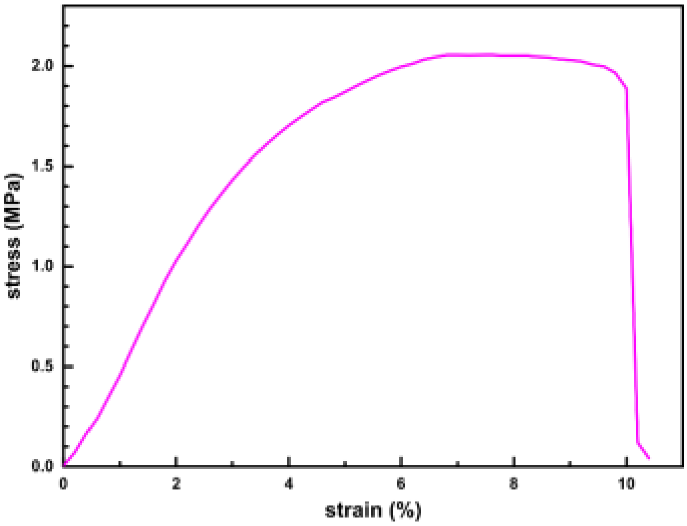

Figure S7. Strain-stress characteristic of the r-PET@Chit2 membrane, which describes the mechanical resistance of the sample.

Table S2. Comparison between the oil-water mixtures separation performances of different amphiphilic membranes with the nanofibrous filter of our design.

Sample	Oil-water mixture	Flux (L m^{-2} h^{-1})	Separation efficiency (%)	Reference
Corn cub powders coated meshes	Hexane-water	~14,400	> 99.9	[1]
	Dichloromethane-water	~21,600	> 99.9	
Waste potato residue coated mesh	Kerosene-water	~25,200	98.5	[2]
	Chloroform-water	~32,400	98.0	
Waste cigarette filter nanofibrous membranes	Kerosene-water	1000	> 99.9	[3]
	Trichloromethane-water	1300	> 99.9	
rPET@Chitosan nanofibrous membrane	Hexane-water	524 ± 75	98.8	This work
	Tetrachloroethylene-water	2074 ± 107	98.1	

Table S3. Values of the viscosity and density for the different liquids used in the filtration tests.

Liquid	Viscosity [mPa s]	Density [g cm^{-3}]
Water	1.00	0.99
Kerosene	1.92	0.79
Hexane	0.31	0.66
Carbon tetrachloride	0.89	1.62
Tetrachloroethylene	0.97	1.59

Table S4. Comparison between the oil-water emulsion separation performances of different amphiphilic membranes with the nanofibrous filter of our design.

Sample	Oil-water emulsion	Flux (L m^{-2} h^{-1})		Separation efficiency (%)		Reference
		Oil in water	Water in oil	Oil in water	Water in oil	
Carbon black-coated membrane	Hexane-water	3500	6500	99.9	99.7	[4]
Fluorinated silica nanoparticles coated paper towel	Hexadecane-water	4480	–	99.9	–	[5]
Waste cigarette filter nanofibrous membranes	Kerosene-water	100	150	99.4	99.7	[3]
rPET@Chitosan nanofibrous membrane	Hexane-water	991 ± 84	233 ± 59	99.9	98.9	This work

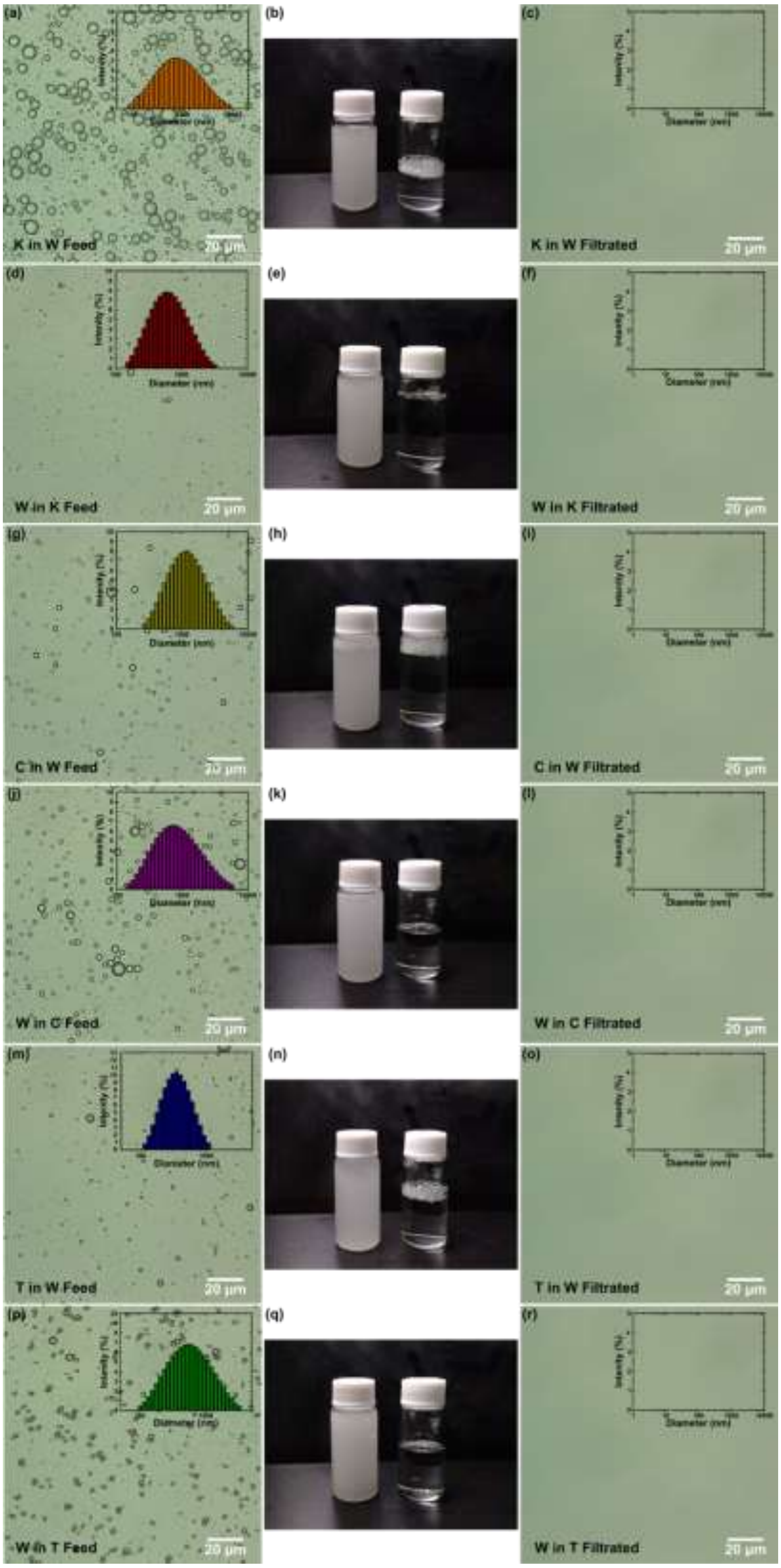

Figure S8. The optical microscope images of (a, g, m) oil in water and (d, j, p) water in oil emulsions together with the corresponding DLS signal. These results are compared with the same analysis operated on the filtrated emulsion (c, f, i, l, o, r). For completeness, the pictures of the emulsions pre and post-filtration are also shown (b, e, h, k, n, q).

References

[1] J. Li, X. Bai, X. Tang, F. Zha, H. Feng, W. Qi, *Sep. Purif. Technol.* **2018**, *195*, 232.
[2] J. Li, D. Li, Y. Yang, J. Li, F. Zha, Z. Lei, *Green Chem.* **2016**, *18*, 541.
[3] W. Liu, M. Cui, Y. Shen, G. Zhu, L. Luo, M. Li, J. Li, *J. Colloid Interface Sci.* **2019**, *549*, 114.
[4] G. Cao, Y. Wang, C. Wang, S.-H. Ho, *J. Mater. Chem. A* **2019**, 11305.
[5] D. Ge, L. Yang, C. Wang, E. Lee, Y. Zhang, S. Yang, *Chem. Commun.* **2015**, *51*, 6149.
